# Genome analysis of the sugar beet pathogen *Rhizoctonia solani* AG2-2IIIB revealed high numbers in secreted proteins and cell wall degrading enzymes

**DOI:** 10.1186/s12864-016-2561-1

**Published:** 2016-03-17

**Authors:** Daniel Wibberg, Louise Andersson, Georgios Tzelepis, Oliver Rupp, Jochen Blom, Lukas Jelonek, Alfred Pühler, Johan Fogelqvist, Mark Varrelmann, Andreas Schlüter, Christina Dixelius

**Affiliations:** Institute for Genome Research and Systems Biology, CeBiTec, Bielefeld University, D-33501 Bielefeld, Germany; Syngenta Seeds AB, Säbyholmsvägen 24, 26191 Landskrona, Sweden; Swedish University of Agricultural Sciences, Department of Plant Biology, Uppsala BioCenter, Linnean Center for Plant Biology, P.O. Box 7080, S-75007 Uppsala, Sweden; Bioinformatics and Systems Biology, Gießen University, D-35392 Gießen, Germany; Institute for sugar beet research, IfZ, Göttingen, Germany

**Keywords:** *Beta vulgaris*, Carbohydrate active enzymes, Carbohydrate esterases, Sugar beet pathogens, Glycoside hydrolases, Polysaccharide lyases, *Rhizoctonia solani*

## Abstract

**Background:**

Sugar beet (*Beta vulgaris*) is a crop cultivated for its high content in sugar, but it is vulnerable to many soil-borne pathogens. One of them is the basidiomycete *Rhizoctonia solani*. This fungal species has a compatibility system regulating hyphal fusions (anastomosis). Consequently, *R. solani* species are categorized in anastomosis groups (AGs). AG2-2IIIB isolates are most aggressive on sugar beet. In the present study, we report on the draft genome of *R. solani* AG2-2IIIB using the Illumina technology. Genome analysis, interpretation and comparative genomics of five sequenced *R. solani* isolates were carried out.

**Results:**

The draft genome of *R. solani* AG2-2IIIB has an estimated size of 56.02 Mb. In addition, two normalized EST libraries were sequenced. In total 20,790 of 21,980 AG2-2IIIB isotigs (transcript isoforms) were mapped on the genome with more than 95 % sequence identity. The genome of *R. solani* AG2-2IIIB was predicted to harbor 11,897 genes and 4908 were found to be isolate-specific. *R. solani* AG2-2IIIB was predicted to contain 1142 putatively secreted proteins and 473 of them were found to be unique for this isolate. The *R. solani* AG2-2IIIB genome encodes a high number of carbohydrate active enzymes. The highest numbers were observed for the polysaccharide lyases family 1 (PL-1), glycoside hydrolase family 43 (GH-43) and carbohydrate estarase family 12 (CE-12). Transcription analysis of selected genes representing different enzyme clades revealed a mixed pattern of up- and down-regulation six days after infection on sugar beets featuring variable levels of resistance compared to mycelia of the fungus grown in vitro.

**Conclusions:**

The established *R. solani* AG2-2IIIB genome and EST sequences provide important information on the gene content, gene structure and transcriptional activity for this sugar beet pathogen. The enriched genomic platform provides an important platform to enhance our understanding of *R. solani* biology.

**Electronic supplementary material:**

The online version of this article (doi:10.1186/s12864-016-2561-1) contains supplementary material, which is available to authorized users.

## Background

Sugar beet (*Beta vulgaris* ssp. *vulgaris*) is a crop grown in temperate geographic regions for its high sugar content in the root, which accounts for about 30 % of the sugar production in the world. This species is one of our youngest cultivated crop plants whereupon work on crop improvements started in the mid-1800s and was enhanced after World War II [1]. Sugar beet has a genome with a size of about 567 Mb comprising 27,421 protein-coding genes [2]. This new knowledge is expected to facilitate further crop improvements and diversified utilization of carbohydrates in various bio-products and for biofuel production. Sugar beets have a rather long growing season that is between 4 to 6 months. Hence, it is vulnerable to many soil-borne pathogens. *Rhizoctonia solani* (telemorph: *Thanatephorus cucumeris*) is one of them. This anamorphic basidiomycete fungus has a wide host range causing disease on more than 200 plant species [3, 4]. On crop plants, *R. solani* is known to cause rice sheath blight, black scurf on potatoes, bare patch on cereals and root or stem rot on soybean among many other diseases [5]. In case of sugar beet, this facultative saprophyte causes crown- and root-rot as well as seedling damping-off resulting in significant damages. The root-rot disease is considered to be most severe inciting losses of 24 % per acre regarding sugar beet cultivation in the US and 10 % in some European regions [6]. The *R. solani* life cycle is not completely known. This fungal species does not produce any asexual spores and only occasionally forms sexual spores. In nature, *R. solani* exists primarily as mycelia and survives as sclerotia in crop residues and soil [6]. These survival structures can reside in soil for many years and germinate and infect sugar beet roots, petioles and crowns under favorable conditions. Typically, leaves collapse and wilt, but often stay attached to the crown. The fungus causes dark lesions on roots, when attacking somewhat older plants; these symptoms develop into rotted root tissue as the disease progresses.

*Rhizoctonia solani* has a compatibility system that regulates the fusion of hyphae. Based on this hyphal reaction, different anastomosis groups (AGs) can be distinguished. At least thirteen AGs have been specified up to now and many of them are further subdivided into so-called inter-specific groups (ISG) featuring different host ranges, culture appearances or thiamine requirements [7–10]. Phylogenetic analysis using 18S rRNA sequences also split the different *R. solani* genotypes into two major clades, suggesting divergent evolution [11]. *R. solani* members of the AG2 group cause canker on many root crops [3]. AG2 is further subdivided into type 1 and type 2. Most isolates of type-2 derive from sugar beets or soils, where sugar beets have been grown. AG2-2 is further subdivided into AG2-2IIIB and AG2-2IV based on pathogenicity and culture morphology characteristics. However, the latter subgroups cannot be distinguished by hyphal fusion. Both subgroups are pathogenic to sugar beet, but AG2-2IIIB isolates are more aggressive than AG2-2IV ones [12]. Here, we report on the 56.02 Mb draft genome of *R. solani* AG2-2IIIB harboring 11,897 predicted genes as determined by application of a specific gene model developed for this isolate. Comparative genomic analyses with four other sequenced *R. solani* isolates representing different anastomosis groups revealed differences regarding predicted secreted proteins and enrichment of cell wall degrading enzymes in *R. solani* AG2-2IIIB.

## Results and discussion

### The *R. solani* AG2-2IIIB draft genome sequence

The draft genome sequence of the *R. solani* AG2-2IIIB isolate BBA 69670 was established by high-throughput sequencing on the Illumina MiSeq system. Sequencing resulted in 14,793,249 reads yielding approximately 2.8 Giga bases of information. Considering the final size of the *R. solani* AG2-2IIIB draft assembly, a 40-fold coverage was estimated. Detailed sequencing statistics of the *R. solani* AG2-2IIIB genome are summarized in Additional file 1: Table S1. After sequencing and assembly of sequencing reads, a ‘contig-length vs. read-count’ analysis was performed to gain deeper insights into *R. solani* AG2-2IIIB genome composition, architecture and partition as previously described [11]. Based on this strategy assembled contigs were classified in five different groups (Additional file 2: Table S2). Group I contigs (lower than 0.5-fold coverage) presumably represent assembly artifacts. Contigs representing groups II (0.5 to 1.5-fold coverage) and III (1.5 to 3-fold coverage) most likely contain chromosomal sequences reflecting the diploid nature of the *R. solani* AG2-2IIIB genome. Group II contigs include sequences that are sufficiently different and therefore were assembled into individual contigs, whereas Group III contigs represent identical or almost identical allelic variants of the sequenced diploid organism. Similar results were obtained for the *R. solani* AG1-IB genome sequence [11]. The contigs of groups IV and V were mostly allocated to the more abundant mitochondrial (mt) genome (group IV, 3 to 50-fold coverage) or the most abundant DNA encoding ribosomal RNAs (group V, > 50-fold coverage). In addition, the CEGMA analysis resulted in identification of 245 of 248 core eukaryotic genes (CEGs) including 231 complete and 14 partial CEGs (Additional file 3: Table S3). The average number of orthologs per CEG was 1.21 (Additional file 3: Table S3). This result underlines the completeness of genome as well as the diploid character of the genome.

The identified *R. solani* AG2-2IIIB mitochondrial (mt) genome consists of eight scaffolds and 157 contigs. Based on this result, the mt genome seems to be fragmented and not completed. Many mt contigs are shorter than 500 bp indicating prevalence of highly repetitive A + T spacer sequences that partly hampered sequencing and assembly. Scaffolds representing the *R. solani* AG2-2IIIB mt genome add up to 125,989 bp featuring a GC content of 35.88 %. Accordingly, the size of the *R. solani* AG2-2IIIB mitochondrial genome appears to be smaller than known mt genomes of other *R. solani* isolates with estimated sizes of approximately 150 kb [11, 13–15]. Core genes, including *nad* gene copies (NADH dehydrogenase subunits), a *cob* gene (cytochrome b), three *cox* genes (cytochrome c oxidase subunits), three *atp* genes (ATP synthase F0 subunits), two ribosomal RNA genes and a ribosomal protein *rbs* gene were predicted on the contigs of this mt genome. In comparison to the *R. solani* AG1-IB mt genome, the *dpoB* gene (coding for a DNA polymerase) is missing in AG2-2IIIB. PCR analysis confirmed the absence of this gene. In addition, 16 sequences encoding tRNAs as well as two LAGLIDADG homing endonucleases were identified. Based on the found conserved mt genes and tRNAs, it appears that the majority of the AG2-2IIIB mt genome was assembled in this approach.

### Development of an AG2-2IIIB-specific gene model based on mapping of Expressed Sequence Tags (ESTs)

In parallel to sequencing of the *R. solani* AG2-2IIIB genome, two normalized EST (cDNA) libraries of the fungal isolate were sequenced. The main objectives of this approach were to elucidate the gene structure of *R. solani* AG2-2IIIB and to deduce a corresponding gene model for improved gene prediction on genomic contigs (scaffolds). ESTs were obtained from *R. solani* AG2-2IIIB grown under two different conditions. Either *R. solani* was cultivated in potato dextrose broth (PDB) or in a sugar beet medium. The latter to enrich for *R. solani* transcripts induced by the presence of particular sugar beet compounds. Moreover, ESTs provide information on precise intron-exon structures. Mapping of ESTs on genomic sequences enables development of a corresponding gene model as previously demonstrated for *R. solani* AG1-IB [16].

Total RNA was isolated after growth of *R. solani* in the media specified above. Subsequently, cDNA libraries were generated and normalized to avoid over-representation of highly expressed transcripts, such as ribosomal RNAs (rRNAs) and transcripts representing ribosomal proteins. Both cDNA libraries were *de novo* sequenced on the Illumina MiSeq platform. This approach yielded 4,605,990 reads and approximately 1.4 Giga bases of sequence information. Detailed results of subsequent *de novo* assemblies by means of the gsAssembler software (version 2.8.) are shown in Additional file 4: Table S4. About 98 % of all reads were assembled indicating saturation of the sequencing approach. Likewise, rarefaction analysis considering formation of isotigs (transcript isoforms) from read sub-samples also showed that ESTs were sequenced in sufficient depth. The largest isotig has a size of 7035 bp, whereas the shortest is 107 bp in size.

Assembled isotigs were mapped onto *R. solani* AG2-2IIIB genome sequences to define the borders of exon-intron and intron-exon junctions, locate gene start-sites and to uncover alternative splicing events (Additional file 4: Table S4). In total, 20,790 of 21,980 *R. solani* AG2-2IIIB isotigs were mapped on the genome with more than 95 % sequence identity and more than 90 % template coverage. A survey of alternative splicing events applying ASTALAVISTA revealed 3903 events. In total, 2634 mapped transcripts represent 'intron-retention' events, 185 'alternative acceptor-site' events, 88 'alternative donor-site' events, 11 'exon-skipping' events, whereas 985 events could not be classified into one of these four categories. Evidence for the definition of correct gene start and stop positions was also deduced from mapped isotigs. Finally, a specific *R. solani* AG2-2IIIB gene model was deduced and applied for gene prediction within the eukaryotic gene prediction program AUGUSTUS as previously described [17]. Development of a specific gene model was motivated by the chance to uncover new genes that are not expressed under the conditions applied for the EST sequencing approach and to enable comparative analyses between the genomes of different *R. solani* isolates.

To further improve gene predictions for the *R. solani* AG2-2IIIB genome, a manual curation step was introduced. To assess the quality of the gene prediction approach, gene products deduced from identified genes were compared with those inferred from ESTs by BLASTP. In total, 95 % of all gene products predicted in the genome were represented by an isotig (19,755 of 20,790), whereas 595 genes were only predicted based on the parameter set. However, 3359 of 19,755 isotigs were mapped twice or more on the genome sequence. Accordingly, the *R. solani* AG2-2IIIB specific gene model proved to be very reliable for gene prediction in this fungal genotype. In total, 11,897 predicted genes were automatically annotated by means of a modified GenDB 2.0 version [18, 19] including about 19 % (2262 genes) that appear as genes with multiple variants. This approach assigned functions and observations with high confidence values to 4204 genes. However, most of the predicted genes were automatically annotated as 'hypothetical' or 'uncharacterized' illustrating insufficient functional characterization of fungal genomes from members of the genus *Rhizoctonia*. The GenDB annotation pipeline successfully assigned 734 gene names, 1,488 EC numbers and 3,565 KOG numbers to the identified *R. solani* AG2-2IIIB genes.

**Fig. 1:**
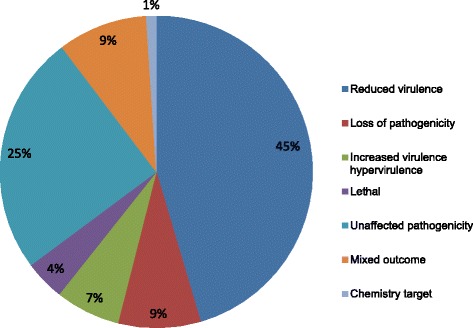
Distribution of phenotypic categories of AG2-2IIIB gene orthologs using the PHI database (www.phi-base.org). The percentage is based on in total 1643 hits

The pathogen-host interaction database (PHI-base) hosts molecular and biological information on more than 2800 pathogen genes, which have been tested experimentally. Out of 1643 hits of the predicted AG2-2IIIB genes, we found the highest proportion (45 %) attributed to the ‘reduced virulence’ category followed by a group of ‘unaffected pathogenicity’ (25 %) as defined by the PHI database [20] (Fig. 1, Additional file 5: Table S5).These assignments probably can be attributed to the wide range of aggressiveness reported between AG groups and isolates and the highly competitive saprophytic ability reported for *R. solani* [21].

### Comparison of *R. solani* genomes with contrasting anastomosis groups

**Fig. 2:**
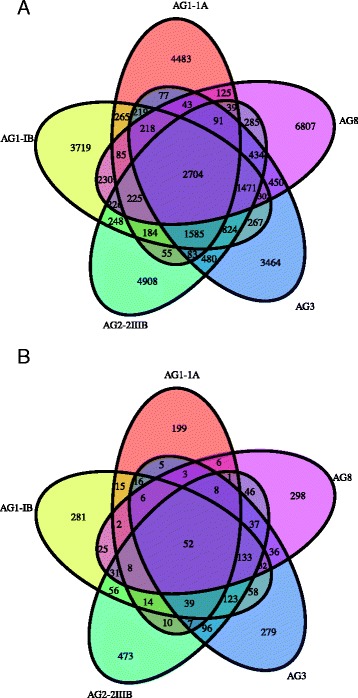
Venn diagrams **a** predicted unique and shared genes and **b** predicted unique and shared secreted proteins using the SignalP tool among five *Rhizoctonia solani* anastomosis groups (AG)

The *R. solani* AG2-2IIIB genome of 56.02 Mbp was compared to those of other sequenced *R. solani* isolates, namely *R. solani* AG1-IA (36.9 Mbp), AG1-IB (42.80 Mbp), AG3 (51.0 Mbp) and AG8 (39.8 Mbp) [11, 14, 15, 17, 22]. For gene-based comparisons of different *R. solani* isolates, the comparative genomics tool EDGAR [23] was applied (Table 1, Additional file 2: Table S2). The core genome of all *R. solani* isolates analyzed consists of 2704 genes representing 19 to 25 % of all genes identified in each isolate (Fig. 2a). *R. solani* AG2-2IIIB draft genome assembly harbors 4908 isolate-specific genes (Fig. 2a). A general feature of *R. solani* is its heterokaryotic and diploid nature, which generates assembly challenges. Various bioinformatics approaches have been used to dissect the ploidy levels of the five genomes sequenced so far. Thus, differences seen between the genomes may to some degree derive from nuclear heterogeneity and precautions should be taken for a too strict interpretation of differences without additional verification.

**Table 1:** Gene predictions in different *R. solani* strains

*R. solani*	No. genes	Average gene size, bp	No. of exons per gene	Average exon size, bp	Average intron size, bp
AG1-1A^a^	10,489	1628	5.78	213.22	83.6
AG1-IB^b^	12,616	1788	6.26	218.71	78.12
AG2-2IIIB	11,897	2245	6.68	221.45	71.23
AG3^c^	12,720	1752	6.47	214.50	66.53
AG8^d^	13,420	1209	4.93	192.79	65.84

^a^Zheng et al. 2013 [14]; ^b^Wibberg et al. 2015 [17]; ^c^Cubeta et al. 2014 [22]; ^d^Hane et al. 2014 [15]

### Analysis of the predicted secretome

**Fig. 3:**
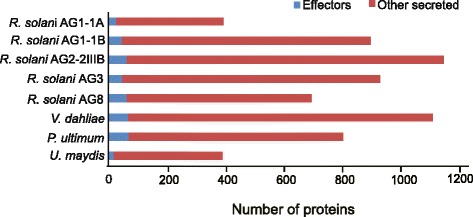
Predicted effectors (*blue*) and other secreted proteins (*red*) in: five *Rhizoctonia solani* anastomosis groups (AGs), two sugar beet pathogens (*Verticillium dahliae* and *Pythium ultimum*) and the basidiomycete species *Ustilago maydis*. Numbers of potential effector proteins (*blue*) are defined as small cysteine-rich proteins featuring at least 3 % cysteine and a maximum size of 400 amino acids

To establish a successful infection and evade plant defense responses during colonization, plant pathogens secrete proteins and other molecules, collectively termed effectors, to various host compartments [24, 25]. These secreted effectors facilitate host colonization and thereby modulate host biochemistry, physiology, and defense responses. Based on the *R. solani* AG2-2IIIB draft genome assembly, 1142 secreted proteins were predicted using the described secretome pipeline [26], and among them, 473 were specific to AG2-2IIIB (Fig. 2b). These results were compared to the other four *R. solani* genomes and to basidiomycete model pathogen *Ustilago maydis* (Fig. 3). The biotrophic fungal group represented by *U. maydis* possesses predicted biotrophic interactors that are highly specialized [27]. This kind of specific functions may not be expected to have evolved in a fungal species like *R. solani* with a broad host range. In general, ascomycetes, particularly necrotrophs thriving on dead or dying plant cells encode high numbers of secreted proteins, here represented by the *Verticillium dahliae*, which is another soilborne pathogen with a broad host range including sugar beets [28]. Further, many fungal effectors are known to be small cysteine-rich proteins with a size of less than 400 amino acids. In each *R. solani* draft genome assembly, around 100 small cysteine-rich proteins are predicted (Table 2; Additional file 6: Table S6). Necrosis and ethylene-inducing-like protein (NLP) genes are common in many plant pathogenic organisms not least those related with wilting disease symptoms like *Fusarium oxysporum* and *V. dahliae* [29, 30]. Thus, NLP-encoding homologues were expected in the *R. solani* genomes but were found absent in all five cases. Another effector group common in particularly ascomycete fungi including *V. dahliae* are genes with a predicted lysine motif (LysM) [31]. However, very few genes harboring LysM were found in the *R. solani* genomes (Table 2). Quantitative real-time PCR (qPCR) was used to determine transcript levels of selected *R. solani* AG2-2IIIB secreted genes. We used four sugar beet breeding lines, two susceptible and two partial resistant towards the AG2-2IIIB isolate to monitor fungal responses upon interaction with its host. First, we checked if the *RSOLAG22IIIB_4067* gene, encoding the putative secreted LysM protein was active during infection. Elevated transcript levels were observed during interactions with the partial resistant genotypes (1 and 2) compared to mycelia grown in vitro, but no significant differences were observed among the plant genotypes (Additional file 7: Figure S1). LysM proteins can be grouped in two classes based on the architecture of the LysM-containing proteins and the location of cysteine residues [32]. Based on present information the *RSOLAG22IIIB_4067* gene has features typical of a fungal-specific LysM motif.

**Table 2:** Predicted secreted proteins in five *R. solani* anastomosis groups (AG)

	AG1-1A	AG1-IB	AG2-2IIIB	AG3	AG8
Isolate origin	Rice	Lettuce	Sugar beet	Potato	Lupin
Total secreted proteins	391	892	1142	925	690
Cysteine-rich proteins^a^	68	115	126	100	133
LysM	1	3	1	2	0
NLP1^b^	0	0	0	0	0

^a^< 400 amino acid, N-terminal signal peptide and cysteine-rich. ^b^Necrosis and ethylene-inducing-like protein

### Distribution of carbohydrate active enzyme genes and transcript analysis of selected gene candidates

**Fig. 4:**
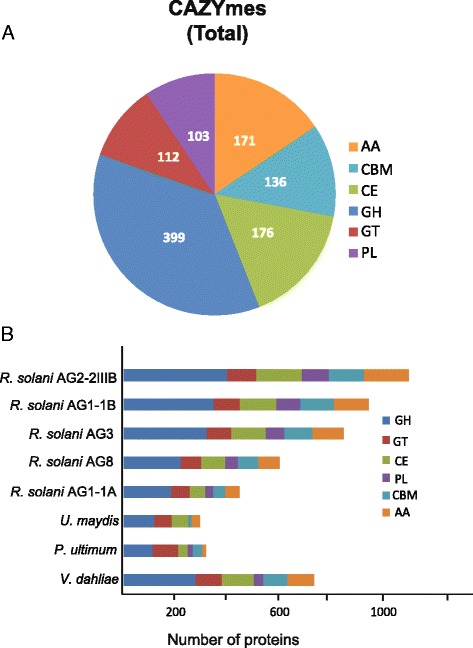
CAZy analysis in *R. solani* AG2-2IIIB. **a** Predicted *R. solani* AG2-2IIIB genes encoding carbohydrate active enzymes as categorized within the CAZy database. **b** Distribution of CAZymes predicted in the genomes of *R. solani* (five anastomosis groups), *Ustilago maydis*, *Pythium ultimum* and *Verticillium dahliae*

The plant cell wall is an important barrier for plants to protect themselves from a range of attacking organisms. Thus, it is essential for a phytopathogenic fungus like *R. solani* AG2-2IIIB, with no specialized penetration appressoria-like structure shown so far, to have means of entering by weakening the host cell walls, thereby promoting colonization. Further, soilborne pathogens are confined within the soil, a complex matrix of minerals, organic matter and a rich diversity of organisms. To survive and multiply, a plant pathogen must compete with a multitude of organisms. Thus, competition for resources, and various community compositions significantly affects pathogen invasion rates on plant roots [33]. In this battlefield, lytic enzymes and secreted toxic compounds are essential [34]. The CAZy database contains enzymes responsible for degrading carbohydrates and glycoconjugates. The LysM domain resides in the CBM50 peptidoglycan-binding module among the carbohydrate active enzymes (CAZymes), a category of enzymes that is able to break down or modify carbohydrate components (pectins, cellulose and hemicellulose) of plant cell walls [35, 36]. CAZymes are common in necrotrophic and saprophytic fungi and have important functions in establishing infection, making nutrients accessible for fungal growth [32]. We categorized the carbohydrate active enzymes encoded in the AG2-2IIIB genome assembly according to the CAZy database and found a large arsenal, in total 1097 predicted plant cell wall-degrading enzymes (Fig. 4a; Additional file 8: Figure S2). Glycoside hydrolases are dominant (399) followed by carbohydrate esterases (176) and auxillary activity enzymes (171) (Fig. 4a). By comparing with *R. solani* AG1A, AG1-1B, AG3 and AG8 data (Fig. 4b), the AG2-2IIIB genome has experienced an expansion and diversification of polysaccharide lyases (PL). This notable enrichment was further accentuated in comparison to the genomes of *U. maydis*, and *Pythium ultimum,* the latter an oomcyete pathogen on sugar beet. *Verticillium dahliae* also features enriched numbers and composition of CAZymes, which has been attributed to its broad host range [30].

Polysaccharides possess multiple functions in all organisms, and are essential components of cell walls of plants where they provide physical rigidity and protection against environmental constrains [37]. Sugar moieties of pectic polysaccharides (arabinose, rhamnose, galactose and galacturonic acid) account for more than 63 % (w/w) of the cell wall material present in sugar beet pulp, and approximately 70 % of the pectin consists of branched rhamnogalacturonans [38]. Further, unlike most dicots, the *Chenopodiaceae* comprise significant levels of ferulic acid in their primary walls [39–41]. Esterases hydrolyze ester bonds and ferulic acid esterases are known to act synergistically with xylanases and pectinases facilitating the access of hydrolases to the backbone of cell wall polymers. Altogether, this could explain why carbohydrate esterases constitute a large proportion of the CAZymes in AG2-2IIIB. *R. solani* in general is known to have a broad host range, but whether occurrence of a particular set of CAZy enzymes determines host range characteristics is still unknown. For example, could the less diverse set of CAZymes encoded in the AG1-1A genome be explained by the fact that this fungal isolate originates from a monocot?

**Fig. 5:**
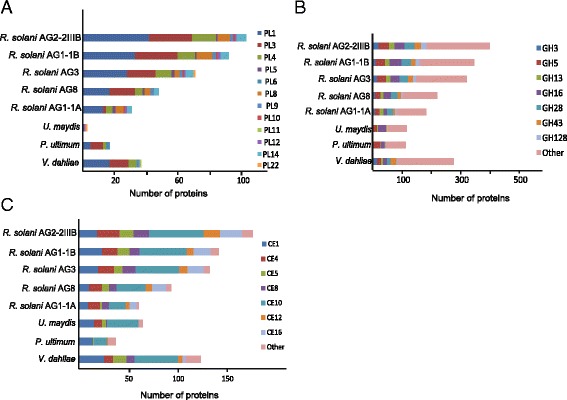
Comparison of CAZyme families identified in *Rhizoctonia solani* (five anastomosis groups), *Ustilago maydis*, *Pythium ulticum* and *Verticillium dahliae*. **a** Polysaccharide lyases (PL), **b** Glysoside hydrolases (GH) and **c** Carbohydrate esterases (CE)

We further looked into the expanded group of polysaccharide lyases (PLs), glycoside hydrolases (GHs), and carbohydrate esterases (CEs), due to their abundance and particular distribution in AG2-2IIIB compared to other *R. solani* genomes (Fig. 4a). A high number of PL1 proteins was observed in AG2-2IIIB (Fig. 5a; Additional file 8: Figure S2). Phylogenetic analysis comprising 33 full length-PLs from the PL1 group that is important for infection and widely distributed among fungi [42] revealed seven clades whereof clade A, B, and F were AG2-2IIIB-specific, with stronger support for clade A and F (Additional file 9: Figure S3a). Elevated levels of the *RSOLAG22IIIB_7799* and *RSOLAG22IIIB_2409* genes (clade F) were found at the compatible interactions compared to control and exposure to the partial resistant genotypes (Additional file 7: Figure S1) indicating a role of this enzyme in pathogenicity. In contrast, the *RSOLAG22IIIB_8439* gene, which was classified to clade B, was down-regulated compared to control samples (Additional file 7: Figure S1). PL15 is common among other fungi of the phylum *Basidiomycota* [42], but is not present in *R. solani* or in the model basidiomycete species *U. maydis*, which only has one PL1 gene. *U. maydis* harbors few CAZymes (81), whereof 38 are predicted to be associated with cell wall modifications [43]. During infection, plant cell wall components also can act as damage-associated molecular patterns that trigger plant defense responses [44]. Thus, it is possible that specific cell wall degrading enzymes such as the enzyme encoded by *RSOLAG22IIIB_8439* is repressed to avoid initiating the plant defense response via release of cell wall fragments.

Glycoside hydrolases are known to catalyze hydrolysis of glycosidic bonds in carbohydrate molecules. To date, 132 GH families have been characterized, and we found that the GH43 family, comprising enzymes acting on pectin and hemicellulose as substrates, is more diverse in *R. solani* AG2-2IIIB compared to other *R. solani* isolates (Fig. 5b). Analysis of 20 full-length GH43 sequences from *R. solani* AG2-2IIIB revealed that they are mainly classified in two clades, I and III (Additional file 9: Figure S3b). We also found that three GH43 members are grouped in clade IV, while four enzymes were categorized in clade V (Additional file 9: Figure S3b). The transcription analysis revealed high expression of the *RSOLAG22IIIB_7342* gene during interactions with the partial resistant genotypes, whereas *RSOLAG22IIIB_2459* encoding a clade V enzyme, was constitutively expressed under our tested conditions (Additional file 7: Figure S1).

As already outlined above, carbohydrate esterases (CE) are enzymes involved in degradation of cellulose and hemicellulose. Our analysis revealed an enrichment of CE12 proteins in AG2-2IIIB (Fig. 5c). Further, the members of the diverse CE12 group were categorized in three distinct clades A, B and C (Additional file 9: Figure S3c). Again the transcription patterns of the CE12 genes, *RSOLAG22IIIB_6451* and *RSOLAG22IIIB_1157* gave ambiguous results (Additional file 7: Figure S1). Finally, distribution and composition of auxiliary activities (AAs), Carbohydrate Binding Modules (CBMs) and Glycosyl Transferases (GTs) in *R. solani* genomes and other soilborne pathogens on sugar beet are presented in Additional file 10: Figure S4.

## Conclusion

The draft genome of *R. solani* AG2-2IIIB encodes a high number of cell wall degrading enzymes. Genes of these categories, which most likely reflects the saprophytic lifestyle of the genus *Rhizoctonia* commonly feeds on dead organic matter of plant origin. The constantly growing genomic information on anastomosis groups and isolates of *R. solani* is an important step to generate better understanding of these important plant pathogens. Development of efficient tools to enable functional analyses in this fungal genus is much awaited to support hypotheses developed from genome interpretation.

## Methods

### Genomic DNA and RNA preparations

The *R. solani* AG2-2IIIB isolate BBA 69670 (DSM 101808) deriving from sugar beet grown in Bavaria, Germany, was used in the study. Fungal mycelia were grown in liquid potato dextrose broth (PDB) in darkness at 23–24 °C for 48 h, under shaking conditions (150 rpm). DNA was extracted using the QIAgen DNAeasy Plant Minikit (Qiagen). For RNA the fungus was grown in PDB or sugar beet media containing 4 g surface-sterilized sugar beet pieces in 100 ml sterile H_2_0 and cultured as above. RNA was isolated by RNeasy kit (Qiagen), and cDNA was prepared at Vertis Biotech, Freising, Germany.

### Genome sequencing and assembly

DNA quality was assessed by gel-electrophoresis and the quantity was estimated by using the Quant-iT PicoGreen dsDNA kit (Invitrogen) and the Tecan Infinite 200 Microplate Reader (Tecan). The draft genome sequence of *R. solani* AG2-2IIIB was established using Illumina MiSeq and mate pair sequencing (2 × 250 bp) with a distance range of about 8 kb. Adapters and low quality reads were removed by an in house software pipeline including CASAVA v 1.8 and other common processing tools, such as FastQC [45] and trimmomatic [46] prior to assembly. A *de novo* assembly was performed using the GS *De Novo* Assembler software version 2.8 with heterozygotic mode and default settings. Resulting genomic contigs were analyzed for large local similarities applying the BLASTn algorithm [47]. The obtained data were used to estimate the number of allelic variants within the *R. solani* AG2-2IIIB genome. In addition, to analyse the genome structure of *R. solani* AG2-2IIIB, a contig-length vs. read-count analysis was performed [11]. The completeness of the assembly was assessed using CEGMA v 2.4 [48].

### Gene prediction and genome annotation

Gene prediction was performed by applying Augustus version 2.6 [49] using default settings with a parameter set based on assembled transcripts of *R. solani* AG2-2IIIIB and *R. solani* AG1-1B [16]*.* Genes were functionally annotated using a modified version of the genome annotation system GenDB 2.0 [19] for eukaryotic genomes as recently described [18]. For automatic annotation, similarity searches against different databases including KOG [50], KEEG [51] and SWISS-PROT [52] were performed. Putative tRNA genes were identified with tRNAscan-SE [53].

### cDNA sequencing, annotation and functional analysis

Two normalized cDNA-shotgun libraries were generated by vertis Biotechnologie AG. Both libraries were sequenced on the Illumina MiSeq system by a half of a flow cell in a paired-end sequencing run (2 × 250 bp) with a distance range of about 500 bp. Adapter sequences were trimmed [16], followed by assembly using the GS *De Novo* Assembler software version 2.8 with default settings for cDNA data sets and quality assessed [54]. The coverage of the constructed normalized cDNA libraries was estimated by a rarefaction analysis as recently described [16] Assumed contaminations, chimeric sequences and sequences with low quality were eliminated. 19,778 isotigs from the PDB dataset and 15,946 isotigs from the sugar beet dataset were used for further analysis. Isogroups, isotigs and contigs were annotated within the Sequence Analysis and Management System (SAMS) 2.0 [55] applying an annotation pipeline consisting of a collection of different standard bioinformatics tools [16]. Functional annotations were created assigning gene names, gene products, EC numbers, GO terms and KOG functional categories. Further functional predictions were done via KEGG, browsing the KOG hierarchy and discovering candidates for functional roles by searching for annotated KOG numbers.

### *Rhizoctonia solani* genome comparisons

Comparisons between the *R. solani* AG2-2IIIB draft genome and the genomes of *R. solani* AG1-IA [14], AG1-IB [11, 17], AG3 [22], and AG8 [15] including identification of orthologous genes and classification of genes as core genes or singletons were accomplished using a modified version of the comparative program EDGAR for eukaryotic genomes with their multi-exon genes [23]. An add-on was implemented into EDGAR that enables import of GenBank-files representing eukaryotic genomes featuring multi-exon genes. Based on this add-on, it is possible to extract encoded protein sequences of the imported GenBank-file.

### Secretome prediction

A pipeline that was recently described [26] including SignalP-4.0 [56] and TMHMM v2.0 [57] was used for the prediction of secreted proteins. Small cysteine rich secreted proteins were defined as having at least 3 % cysteine and being maximum 400 amino acids long. Enrichment of Pfam domains in the set of predicted secretome was calculated as in Chandran et al. [58].

### Identification and analysis of carbohydrate-related proteins

Carbohydrate active enzymes (CAZymes) in the protein models of *R. solani* were analyzed with dbCAN “DataBase for automated Carbohydrate-active enzyme Annotation” annotation pipeline [59]. The protein models for all other compared organisms were analyzed equally. CAZy-family definitions were according to the CAZy database (http://www.cazy.org/) [60]. The five *R. solani* genomes were compared with *Ustilago maydis* a biotroph basidiomycete [61], and two sugar beet pathogens, the ascomycete *Verticillium dahliae* [30], and the oomycete *Pythium ultimum* [62].

### Gene family phylogenetic analysis

*Rhizoctonia solani* PL1, GH43 and CE12 amino acid sequences and corresponding sequences derived from *Botrytis cinerea*, *Aspergillus nidulans*, *V. dahliae, Magnaporthe oryzae*, *Fusarium graminearum* genomes were aligned with the Clustal W software [63], while sequences from *Arabidopsis thaliana* and *Bacillus* sp., were used as outgroups to improve node support. Phylogenetic analyses were based on catalytic domains predicted by the SMART protein tool [64], and InterProScan [65]. Full-length gene models were carried out using the maximum likelihood method implemented in the MEGA v.6.06 software [66]. The WAG (for PL1 analysis) and WAG + F (for GH43 and CE12 analysis) substitution models were used [67]. Bootstrap analyses were performed using 1000 replicates. Phylogenetic analysis using protein sequences derived from the other four *R. solani* genomes generated extremely low node support and was deleted from further analysis.

### Quantitative real-time PCR

qPCR analyses were performed on infected-plant samples. Total RNA was extracted from partial resistant and susceptible sugar beet seedlings (Additional file 11: Figure S5) inoculated with *R. solani* AG2-2IIIB. 14-days old seedlings were inoculated by putting four infected millet seeds in the soil around each seedling. 6-days post inoculation 1cm of the hypocotyls from 15 seedlings per genotype were sampled and directly put in RNA storage solution following manufacturer’s recommendations (Sigma-Aldrich) and stored in -80^o^C until use.  RNA from *R. solan*i mycelium grown on PDB for 6 days was used as a control. At least four biological replicates, each containing materials from three independent seedlings, were prepared for each plant genotype. RNA extraction was carried out using Qiagen RNeasy Plant Mini Kit (Qiagen) and cDNA synthesis with qScript^™^ cDNA synthesis kit (Quanta Bioscicences). The following program was used: 95 °C for 5 min, 40 cycles of 95 °C for 30 s, 58 to 60 °C for 1 min and 72 °C for 30 s. Primers were designed using the PrimerSelect software implemented in the Lasergene 9 core suite package (DNAstar, Madison, WI). They were designed to amplify 100–165 bp from predicted exons. Primer specificity was tested using non-infected seedlings, where no amplification was observed, and optimal annealing temperature was evaluated using gradient PCR. The primers used in this study are listed in Additional file 12: Table S7. Expression of genes was normalized by *G3PDH* expression [68] and relative expression values were calculated according to the 2^-∆∆Ct^ method [69]. Analysis of variance (ANOVA, one way) was conducted on gene expression and phenotypic data using a General Linear Model implemented in SPSS ver. 20 (IBM, Armonk, NY). Pairwise comparisons were performed using the Tukey’s method at the 95 % significance level.

### Availability of supporting data

All sequence data is available at the DDBJ/EMBL/GenBank database under the accession numbers CYGV01000001 - CYGV01002065. Further information can be found in Wibberg et al. 2016 [70].

## Additional files

Additional file 1: Table S1. Assembly statistics of *R. solani* AG2-2IIIB draft genome. (DOCX 44 kb) Additional file 2: Table S2. Statistics of the contig-length vs. read-count analysis. (DOCX 19 kb) Additional file 3: Table S3. Statistics of the completeness of the genome based on 248 core eukaryotic genes (CEGs). (DOC 25 kb) Additional file 4: Table S4. Sequencing and assembly statistics of *R. solani* AG-2IIIB EST datasets. (DOCX 50 kb) Additional file 5: Table S5. Distribution of phenotypic categories of *R. solani* AG2-2IIIB gene orthologs using the PHI database. (XLS 290 kb) Additional file 6: Table S6. Small cysteine-rich effector proteins with a size of less than 400 amino acids predicted for 5 *R. solani* AGs, *Ustilago maydis*, *Verticillium dahliae* and *Pythium ultimum*. (DOCX 108 kb) Additional file 7: Figure S1. Expression profiles of selected *R. solani* AG2-2IIIB genes during infection of sugar beet seedlings 6 dpi. Genotypes 1 and 2 are partial resistant, while genotypes 3 and 4 are susceptible. Bars represent mean ± SD on at least 4 biological replicates. Different letters (a, b, c) indicate statistically significant differences (*P* ≤ 0.05) using the Tukey test (PDF 435 kb) Additional file 8: Figure S2. Distribution of CAZyme gene families of *R. solani* AG2-2IIIB within 6 main categories according to the CAZy database. Glysoside hydrolases (GH), Carbohydrate esterases (CE), Auxilliary activity (AA), Carbohydrate-binding modules (CBM), Glycosyl transferases (GT), and Polysaccharide lyases (PL). (PDF 1185 kb) Additional file 9: Figure S3. Gene family phylogeny of (a) PL1, (b) GH43 and (c) CE12. Analysis was conducted using the maximum likelihood method with the WAG (for PL1) or WAG + F (for GH43 and CE12) substitution model based on CLUSTAL W alignments of catalytic domain amino acid sequences and 1000 bootstraps. Numbers at nodes indicate the bootstrap value. The protein ID accession numbers originate from the respective database entry. *R. solani* AG2-2IIIB proteins are indicated in red. (PDF 970 kb) Additional file 10: Figure S4. Number of CAZymes identified in *Rhizoctonia solani* (five anastomosis groups), *Ustilago maydis*, *Pythium ulticum* and *Verticillium dahliae*. (a) Auxiliary Activities (AA), (b) Carbohydrate Binding Modules (CBM) and (c) Glycosyl Transferases (GT). (PDF 405 kb) Additional file 11: Figure S5. Phenotypic assessment of *R. solani* AG2-2IIIB infection on roots of four sugar beet genotypes. 13-weeks old plants, were inoculated with *R. solani* AG2-2IIIB by putting 4 infected barley kernels approximately 1 cm from the root and 1.5 cm down in the soil on 4 sides of each root. The graph shows estimated percentage of the root surface covered by necrosis in relation to healthy tissue. At least three roots for each time-point were scored. The observations are presented as average values (Y-axis). (PDF 314 kb) Additional file 12: Table S7. Primer sequences used in the current study. (DOCX 86 kb)

## Abbreviations

AA: auxiliary activities
AG: anastomosis group
CAZy: carbohydrate active enzymes
CBM: carbohydrate binding modules
CE: carbohydrate esterase
CEG: core eukaryotic gene
COG: cluster of orthologous gene
DPI: days post inoculation
EC: enzyme commission
EST: expressed sequence tag
GH: glycoside hydrolase
GO: gene ontology
GT: glycosyl transferase
ISG: inter-specific group
KEGG: Kyoto encyclopedia of genes and genomes
NLP: Necrosis and ethylene-inducing-like protein
PDB: potato dextrose broth
PHI: pathogen host interaction
PL: polysaccharide lyase
SAMS: sequence analysis and management system
