# Supplementary material for: Genome analysis of the sugar beet pathogen Rhizoctonia solani AG2-2IIIB revealed high numbers in secreted proteins and cell wall degrading enzymes
Source: BMC Genomics. 2016 Mar 17;17:245. doi: 10.1186/s12864-016-2561-1 (PMC4794925; doi:10.1186/s12864-016-2561-1)
Supplement: Additional file 1: Table S1. — Assembly statistics of R. solani AG2-2IIIB draft genome. (DOCX 44 kb) [file 12864_2016_2561_MOESM1_ESM.docx]

| **Sequencing method** | Illumina Miseq: 250 bp mate-paired, 8 kb insert size |
| --- | --- |
| **Assembly method** | GS De Novo Assembler 2.8 |
| **Isolate** | AG2-2IIIB |
| **Total assembly length** | 56.02 Mbp |
| **Average scaffold length** | 27,132 bp |
| **Maximum scaffold length** | 1,159,956 bp |
| **Minimum scaffold length** |  |
| **N50** | 143 |
| **N50 length** | 81,152 bp |
| **Total number of scaffolds** | 2,065 |
| **Protein-coding genes** | 11,897 |
| **Predicted tRNA genes** | 135 |

**Table S1.** Assembly statistics of *R. solani* AG2-2IIIB draft genome
