# Supplementary material for: Genome analysis of the sugar beet pathogen Rhizoctonia solani AG2-2IIIB revealed high numbers in secreted proteins and cell wall degrading enzymes
Source: BMC Genomics. 2016 Mar 17;17:245. doi: 10.1186/s12864-016-2561-1 (PMC4794925; doi:10.1186/s12864-016-2561-1)
Supplement: Additional file 2: Table S2. — Statistics of the contig-length vs. read-count analysis. (DOCX 19 kb) [file 12864_2016_2561_MOESM2_ESM.docx]

**Table S2.** Statistics of the contig-length vs. read-count analysis

|  | **Group I^a^** | **Group II^a^** | **Group III^a^** | **Group IV^a^** | **Group V^a^** |
| --- | --- | --- | --- | --- | --- |
| **Ratio^b^** | < 0.5x | 0.5x – 1.5x | 1.5x – 3x | 3x - 50x | > 50x |
| **Total number of contigs** | 217 | 21,232 | 11,607 | 4454 | 64 |
| **^c^Local similarities within these contigs**  **(> 1000 bp)** | 0 | 8,567 | 65 | 9 | 0 |
| **Largest contig** | 2046 bp | 49,565 bp | 41,247 bp | 16,872 bp | 2071 bp |
| **Percentage of all contigs** | 0.5% | 56.72% | 30.87% | 11.84% | 0.1% |
| **Total number of reads** | 1957 | 8,076,982 | 16,954,542 | 1,728,354 | 176,534 |
| **Total contig length** | 46,781 bp | 55,484,562 bp | 13,132,397 bp | 1,809,245 bp | 18,599 bp |
| **Average contig length** | 215 bp | 2613 bp | 1131 bp | 406 bp | 291 bp |

^a^ Contigs were subdivided into groups based on the contig-length vs. read-count plot analysis.

^b^ Ratios were calculated using the equations described by Wibberg et al. [11].

^c^Contigs with a length >1000 bp were searched for local similarities to other contigs within their group. The table shows the number of regions that feature a high similarity (>90%) to contigs of the same group.
