# Supplementary material for: Genome analysis of the sugar beet pathogen Rhizoctonia solani AG2-2IIIB revealed high numbers in secreted proteins and cell wall degrading enzymes
Source: BMC Genomics. 2016 Mar 17;17:245. doi: 10.1186/s12864-016-2561-1 (PMC4794925; doi:10.1186/s12864-016-2561-1)
Supplement: Additional file 3: Table S3. — Statistics of the completeness of the genome based on 248 core eukaryotic genes (CEGs). (DOC 25 kb) [file 12864_2016_2561_MOESM3_ESM.doc]

**Table S3**. Statistics of the completeness of the genome based on 248 CEGs

**Prots^1^  %Completeness^2^ Total^3^ Average^4^  %Ortho^5^**

**Complete** 231 93.14 280 1.21 19.35

**Group 1** 61 92.42 75 1.23 21.21

**Group 2** 53 94.64 63 1.19 16.07

**Group 3** 54 87.09 68 1.26 22.58

**Group 4** 63 96.92 74 1.17 16.92

**Partial** 245 98.79 296 1.21 19.75

**Group 1** 64 96.97 78 1.22 21.21

**Group 2** 55 98.21 65 1.18 17.58

**Group 3** 62 100.00 76 1.23 22.58

**Group 4** 64 98.46 77 1.20 16.92

These results are based on the set of genes selected by Genis Parra ^1^ Prots = number of 248 ultra-conserved CEGs present in genome ^2^ %Completeness = percentage of 248 ultra-conserved CEGs present ^3^Total = total number of CEGs present including putative orthologs ^4^Average = average number of orthologs per CEG ^5^ %Ortho = percentage of detected CEGS that have more than 1 ortholog
