# Supplementary material for: Genome analysis of the sugar beet pathogen Rhizoctonia solani AG2-2IIIB revealed high numbers in secreted proteins and cell wall degrading enzymes
Source: BMC Genomics. 2016 Mar 17;17:245. doi: 10.1186/s12864-016-2561-1 (PMC4794925; doi:10.1186/s12864-016-2561-1)
Supplement: Additional file 4: Table S4. — Sequencing and assembly statistics of R. solani AG-2IIIB EST datasets. (DOCX 50 kb) [file 12864_2016_2561_MOESM4_ESM.docx]

| **Table S4.** Sequencing and assembly statistics of *R. solani* AG2-2IIIB EST datasets | | |
| --- | --- | --- |
| **Features** | **Grown in potato dextrose broth medium** | **Grown in sugar beet**  **medium** |
| **Sequenced reads** | 2,489,643 | 2,116,347 |
| **Sequenced bases** | 735,966,262 bp | 631,513,112 bp |
| **Assembled reads** | 2,441,710 (98%)* | 2,072,237 (97%)* |
| **Total number of assembled bases** | 716,278,249 bp (97%)* | 614,436,880 bp (97%)* |
| **Genes (Isogroups)** | 12,417 | 11,566 |
| **Isoforms of transcripts (Isotigs)** | 19,743 | 15,934 |
| **Contigs:** | 22,354 | 18,411 |
| **GC content** | 51.35% | 51.73% |
| **Hypothetical protein** | 12,417 | 10,118 |
| **Assigned function** | 7,104 | 5,816 |
| **KOG** | 5,139 | 4,051 |
| **KEGG** | 4,265 | 3,192 |
|  |  |  |

* Percentage of the complete bases/reads for each dataset.
