# Supplementary material for: Genome analysis of the sugar beet pathogen Rhizoctonia solani AG2-2IIIB revealed high numbers in secreted proteins and cell wall degrading enzymes
Source: BMC Genomics. 2016 Mar 17;17:245. doi: 10.1186/s12864-016-2561-1 (PMC4794925; doi:10.1186/s12864-016-2561-1)
Supplement: Additional file 6: Table S6. — Small cysteine-rich effector proteins with a size of less than 400 amino acids predicted for 5 R. solani AGs, Ustilago maydis, Verticillium dahliae and Pythium ultimum. (DOCX 108 kb) [file 12864_2016_2561_MOESM6_ESM.docx]

| **Table S6**. Small cysteine-rich effector proteins with a size of less than 400 amino acids predicted for 5 *R. solani* AGs, *Ustilago maydis*, *Verticillium dahliae* and *Pythium ultimum*. | | | | | | | | |
| --- | --- | --- | --- | --- | --- | --- | --- | --- |
|  | **AG1-1A** | **AG1-1B** | **AG2-2IIIB** | **AG3** | **AG8** | ***U. maydis*** | ***V. dahliae*** | ***P. ultimum*** |
| acetyl xylan esterase | 0 | 0 | 0 | 0 | 0 | 0 | 2 | 0 |
| allergen protein | 1 | 1 | 0 | 1 | 2 | 1 | 0 | 0 |
| alpha beta-hydrolase | 0 | 0 | 1 | 0 | 0 | 0 | 0 | 0 |
| b chain crystal structure | 0 | 0 | 0 | 0 | 0 | 0 | 0 | 1 |
| beta-glucan synthesis-associated | 0 | 0 | 0 | 0 | 0 | 0 | 0 | 1 |
| biotrophy-associated secreted protein 2 | 0 | 0 | 0 | 0 | 0 | 0 | 1 | 0 |
| calcineurin-like phosphoesterase | 0 | 0 | 0 | 0 | 0 | 0 | 0 | 1 |
| carbohydrate esterase | 0 | 0 | 0 | 1 | 0 | 0 | 1 | 0 |
| carbohydrate-binding | 0 | 1 | 0 | 1 | 2 | 0 | 0 | 1 |
| cell wall protein | 0 | 0 | 0 | 0 | 0 | 0 | 1 | 0 |
| cellulose binding elicitor lectin | 0 | 0 | 0 | 0 | 0 | 0 | 0 | 1 |
| cfem domain protein (fungal pathogenesis) | 1 | 3 | 6 | 4 | 3 | 0 | 3 | 0 |
| chat domain protein | 0 | 0 | 1 | 0 | 0 | 0 | 0 | 0 |
| chitin binding protein | 0 | 2 | 2 | 1 | 0 | 0 | 0 | 0 |
| choline transport protein | 0 | 0 | 1 | 0 | 0 | 0 | 0 | 0 |
| collagen-like protein mcl1 | 0 | 0 | 0 | 0 | 0 | 0 | 1 | 0 |
| cop8 partial | 0 | 2 | 1 | 1 | 1 | 0 | 0 | 0 |
| cutinase | 0 | 0 | 1 | 0 | 0 | 0 | 0 | 0 |
| cysteine protease family | 0 | 0 | 0 | 0 | 0 | 0 | 0 | 1 |
| cysteine-rich protein | 0 | 0 | 0 | 0 | 0 | 0 | 0 | 1 |
| dehydrogenase | 0 | 2 | 2 | 0 | 0 | 1 | 0 | 1 |
| elicitin-like protein | 0 | 0 | 0 | 0 | 0 | 0 | 0 | 5 |
| endoglucanase | 0 | 0 | 1 | 1 | 0 | 0 | 1 | 0 |
| epl1 protein | 0 | 0 | 0 | 0 | 0 | 0 | 2 | 0 |
| expansin | 0 | 0 | 0 | 0 | 1 | 0 | 0 | 0 |
| extracellular cystatin-like protease inhibitor | 0 | 0 | 0 | 0 | 0 | 0 | 0 | 1 |
| extracellular membrane protein | 0 | 0 | 0 | 0 | 0 | 0 | 1 | 0 |
| fad-binding domain protein | 0 | 0 | 0 | 0 | 2 | 0 | 0 | 0 |
| filamentous hemagglutinin | 0 | 0 | 0 | 0 | 0 | 0 | 1 | 0 |
| fruiting body protein sc7 | 0 | 0 | 0 | 0 | 1 | 0 | 0 | 0 |
| fungal cellulose binding | 1 | 0 | 0 | 0 | 0 | 0 | 1 | 0 |
| gcc2 and gcc3 family protein | 0 | 1 | 1 | 0 | 0 | 0 | 0 | 0 |
| gdsl-like lipase acylhydrolase | 0 | 0 | 0 | 0 | 1 | 0 | 0 | 0 |
| gliding motility protein | 0 | 0 | 0 | 0 | 0 | 0 | 0 | 1 |
| glycopeptide protein | 0 | 1 | 3 | 2 | 1 | 0 | 0 | 0 |
| glycosidase | 1 | 0 | 0 | 0 | 0 | 0 | 0 | 0 |
| glycoside hydrolase | 0 | 1 | 3 | 3 | 2 | 0 | 1 | 1 |
| gpi-anchored leucine-rich lipoprotein | 0 | 0 | 0 | 0 | 0 | 0 | 0 | 2 |
| growth factor receptor | 0 | 0 | 1 | 0 | 0 | 0 | 0 | 0 |
| helicase domain-containing protein | 0 | 0 | 0 | 1 | 0 | 0 | 0 | 0 |
| hydrolytic enzyme protein | 0 | 0 | 0 | 0 | 0 | 0 | 1 | 0 |
| hydrophobin | 0 | 0 | 1 | 0 | 0 | 1 | 3 | 0 |
| hypothetical protein/unknown | 16 | 13 | 17 | 7 | 24 | 9 | 32 | 37 |
| indigoidine synthase a-like protein | 1 | 0 | 0 | 0 | 0 | 0 | 0 | 0 |
| kazal-type proteinase inhibitor | 0 | 0 | 0 | 0 | 0 | 0 | 0 | 2 |
| keratin-associated protein 10-4 | 0 | 0 | 0 | 0 | 0 | 1 | 0 | 0 |
| lipase atg15 | 1 | 0 | 0 | 0 | 0 | 0 | 0 | 0 |
| lipocalin-like domain-containing protein | 0 | 0 | 0 | 0 | 0 | 0 | 1 | 0 |
| lysozyme | 0 | 0 | 0 | 0 | 0 | 0 | 1 | 0 |
| mastigonemelike protein | 0 | 0 | 0 | 0 | 0 | 0 | 0 | 1 |
| med17 domain-containing protein | 0 | 1 | 1 | 1 | 0 | 0 | 0 | 0 |
| metalloprotease | 0 | 0 | 0 | 0 | 0 | 0 | 1 | 0 |
| mmc protein | 0 | 0 | 0 | 0 | 0 | 0 | 1 | 0 |
| non-catalytic module family expn protein | 0 | 0 | 1 | 0 | 1 | 0 | 0 | 0 |
| nuclease pa3 | 0 | 0 | 0 | 0 | 0 | 0 | 1 | 0 |
| nucleoside triphosphate hydrolase | 0 | 0 | 0 | 1 | 0 | 0 | 0 | 0 |
| oxidoreductase | 0 | 1 | 0 | 0 | 0 | 0 | 1 | 0 |
| pectate | 0 | 0 | 0 | 0 | 1 | 0 | 0 | 0 |
| pectate lyase | 0 | 0 | 1 | 2 | 0 | 0 | 2 | 1 |
| pectin lyase | 0 | 0 | 0 | 0 | 0 | 0 | 1 | 0 |
| peptidoglycan-binding | 0 | 0 | 1 | 1 | 0 | 0 | 0 | 0 |
| potassium ion channel yvc1 | 0 | 0 | 0 | 0 | 0 | 1 | 0 | 0 |
| pre-rrna processing protein | 0 | 0 | 0 | 0 | 1 | 0 | 0 | 0 |
| pria protein | 0 | 2 | 3 | 2 | 1 | 0 | 0 | 0 |
| probable inorganic polyphosphate atp-nad kinase | 0 | 0 | 0 | 0 | 0 | 1 | 0 | 0 |
| proline-rich protein | 0 | 1 | 1 | 1 | 0 | 0 | 0 | 0 |
| proteasome regulatory subunit 12 | 0 | 1 | 0 | 0 | 2 | 0 | 0 | 0 |
| protein serine threonine | 0 | 0 | 1 | 0 | 0 | 0 | 0 | 0 |
| protein tos1 | 0 | 0 | 0 | 0 | 0 | 0 | 0 | 1 |
| rare lipoprotein a | 0 | 0 | 0 | 0 | 3 | 0 | 1 | 0 |
| ribonuclease | 0 | 1 | 0 | 3 | 1 | 1 | 1 | 1 |
| scp-like extracellular protein | 0 | 0 | 0 | 0 | 0 | 0 | 0 | 2 |
| sec14 cytosolic factor (transport) | 0 | 0 | 1 | 0 | 0 | 0 | 0 | 0 |
| sig3-like protein | 0 | 0 | 0 | 0 | 0 | 0 | 0 | 1 |
| starch binding domain-containing protein | 0 | 0 | 0 | 0 | 0 | 0 | 1 | 0 |
| sterol uptake control protein 2 | 0 | 0 | 0 | 0 | 0 | 0 | 1 | 0 |
| tenascin-like protein | 0 | 0 | 0 | 0 | 0 | 0 | 0 | 1 |
| teneurin-3-like isoform x6 | 0 | 0 | 0 | 0 | 0 | 0 | 0 | 1 |
| thaumatin-like protein | 3 | 5 | 6 | 6 | 5 | 0 | 0 | 0 |
| tkl protein kinase | 0 | 0 | 0 | 0 | 0 | 0 | 0 | 2 |
| transferase | 0 | 0 | 0 | 0 | 1 | 0 | 0 | 0 |
| transmembrane protein | 0 | 1 | 1 | 2 | 3 | 0 | 0 | 0 |
| wsc domain protein | 0 | 2 | 0 | 3 | 1 | 0 | 0 | 0 |
| xylanase | 0 | 1 | 0 | 0 | 0 | 0 | 0 | 0 |
