# Supplementary figures and images for: Genome analysis of the sugar beet pathogen Rhizoctonia solani AG2-2IIIB revealed high numbers in secreted proteins and cell wall degrading enzymes
Source: BMC Genomics. 2016 Mar 17;17:245. doi: 10.1186/s12864-016-2561-1 (PMC4794925; doi:10.1186/s12864-016-2561-1)

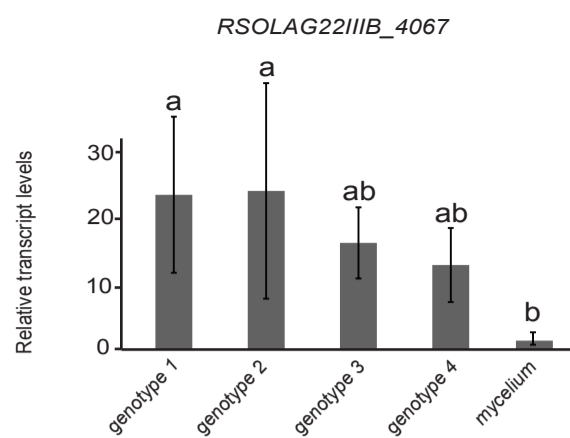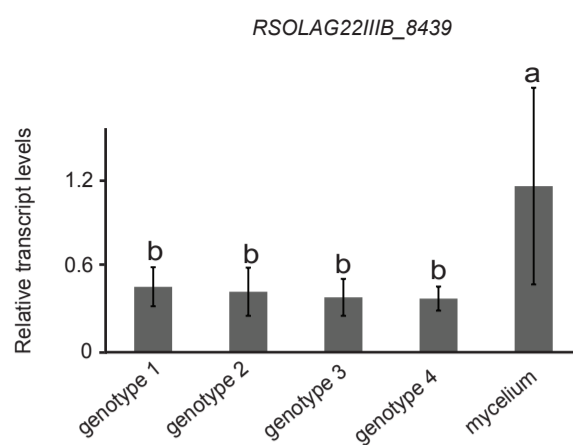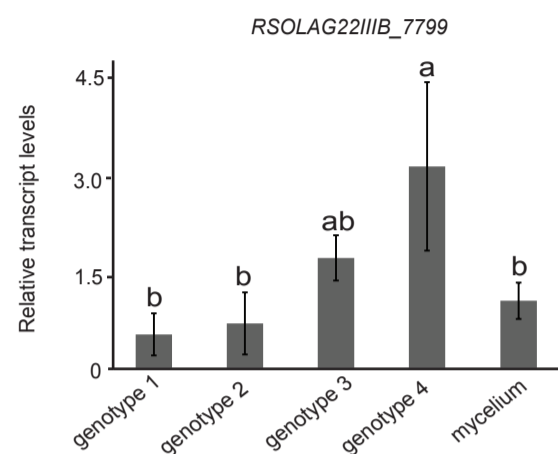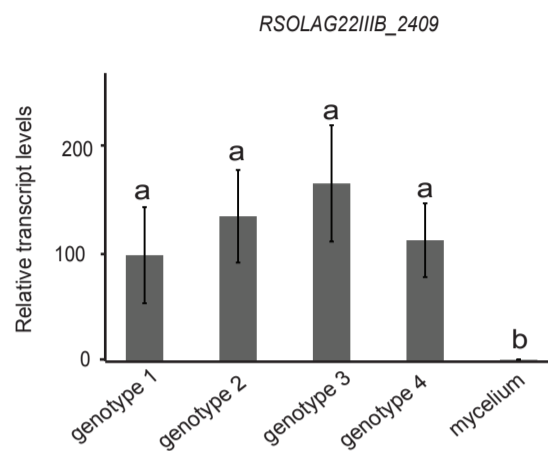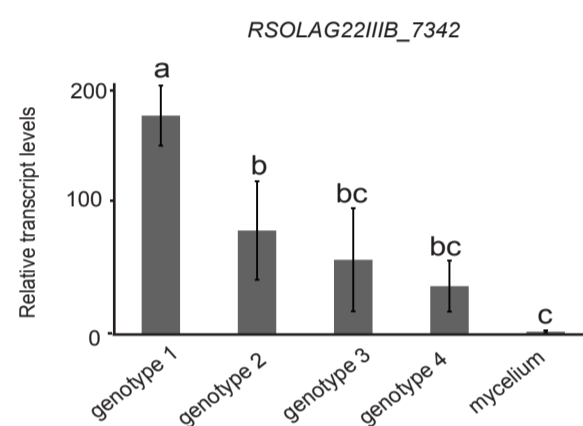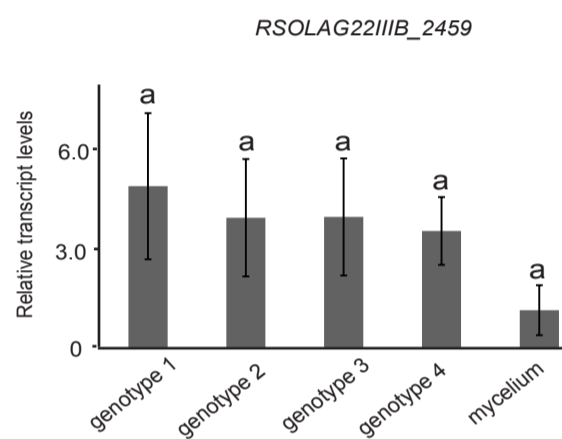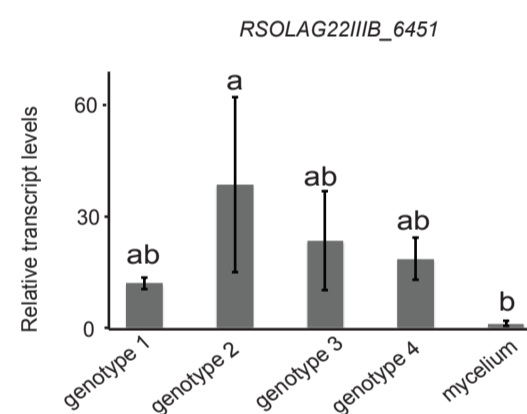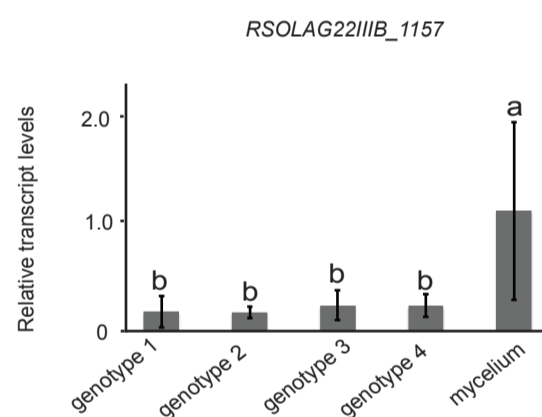

Supplement: Additional file 7: Figure S1. — Expression profiles of selected R. solani AG2-2IIIB genes during infection of sugar beet seedlings 6 dpi. Genotypes 1 and 2 are partial resistant, while genotypes 3 and 4 are susceptible. Bars represent mean ± SD on at least 4 biological replicates. Different letters (a, b, c) indicate statistically significant differences (P ≤ 0.05) using the Tukey test (PDF 435 kb) [file 12864_2016_2561_MOESM7_ESM.pdf]

A

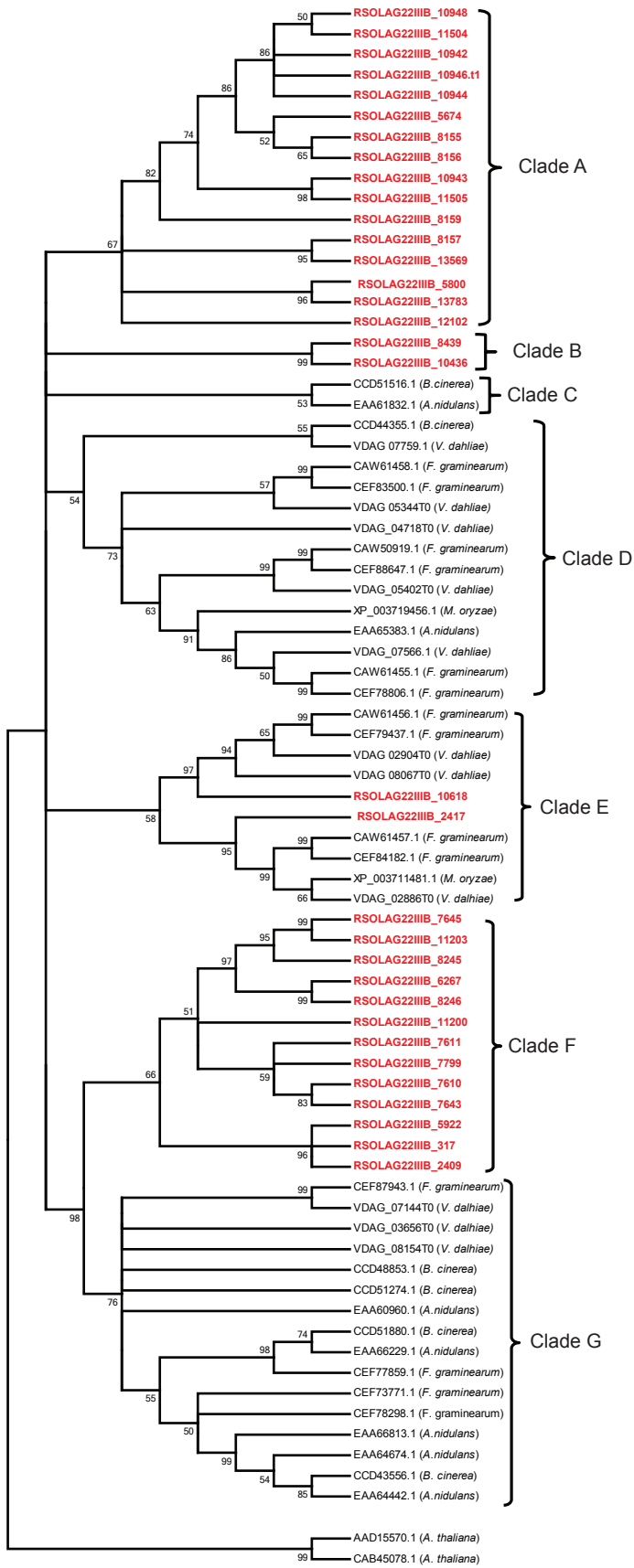

B

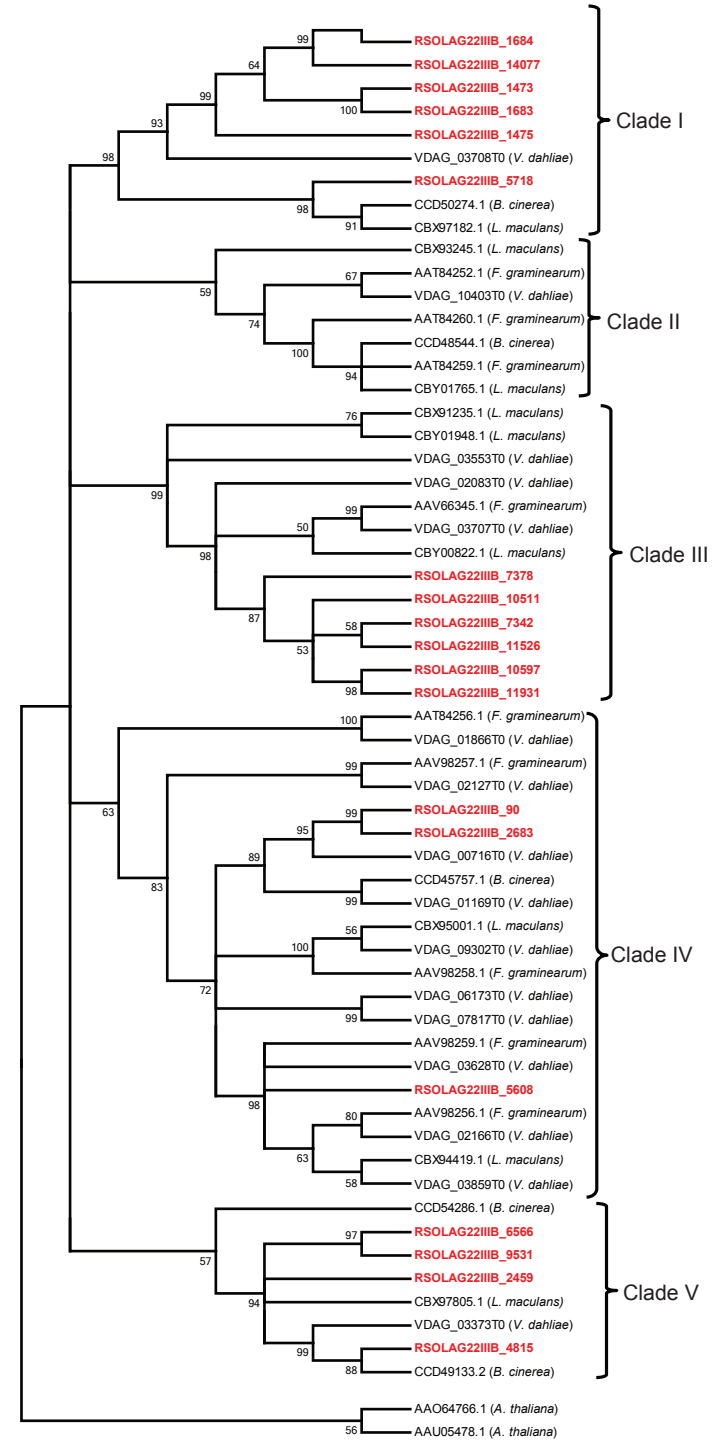

C

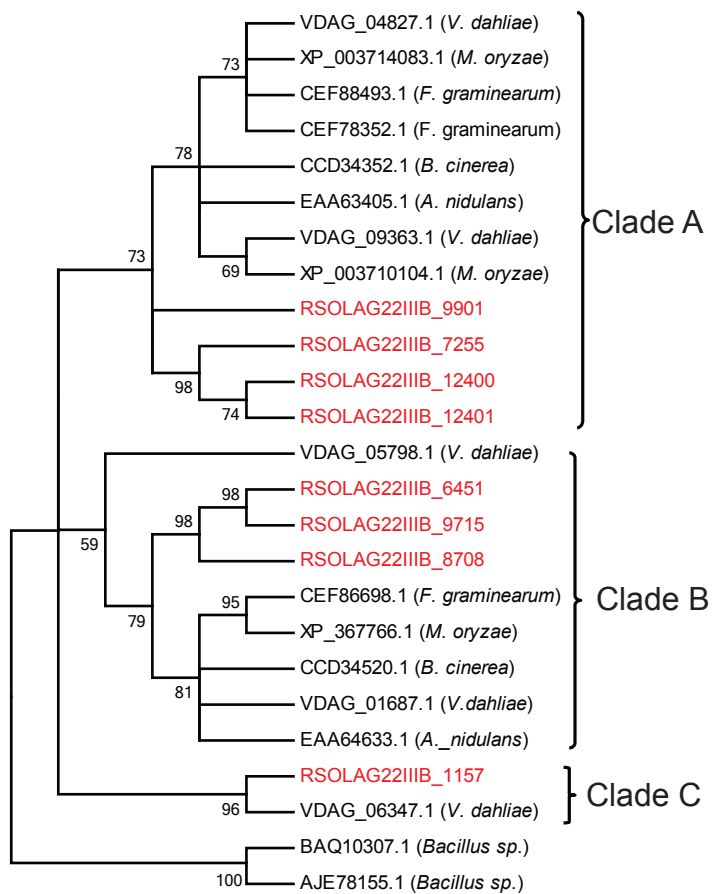

Supplement: Additional file 9: Figure S3. — Gene family phylogeny of (a) PL1, (b) GH43 and (c) CE12. Analysis was conducted using the maximum likelihood method with the WAG (for PL1) or WAG + F (for GH43 and CE12) substitution model based on CLUSTAL W alignments of catalytic domain amino acid sequences and 1000 bootstraps. Numbers at nodes indicate the bootstrap value. The protein ID accession numbers originate from the respective database entry. R. solani AG2-2IIIB proteins are indicated in red. (PDF 970 kb) [file 12864_2016_2561_MOESM9_ESM.pdf]

A

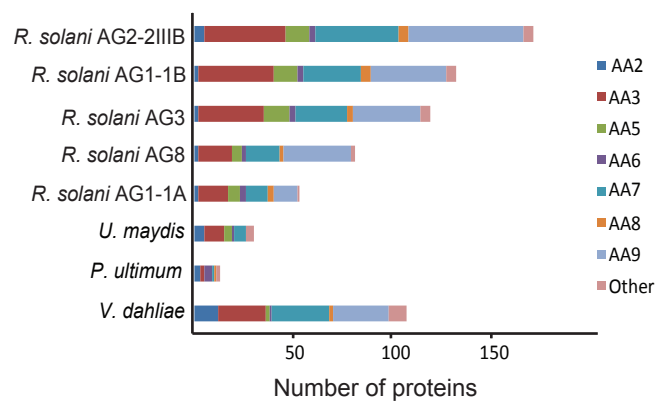

B

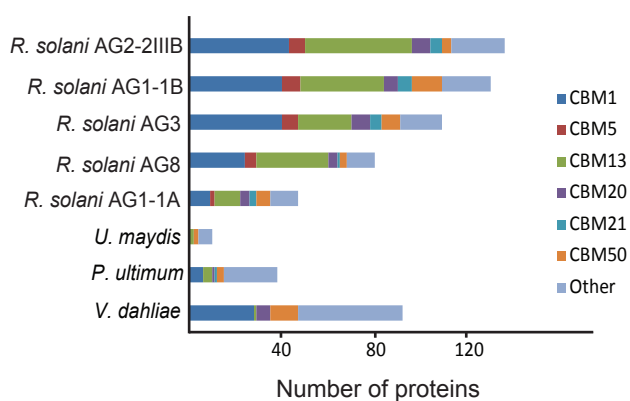

C

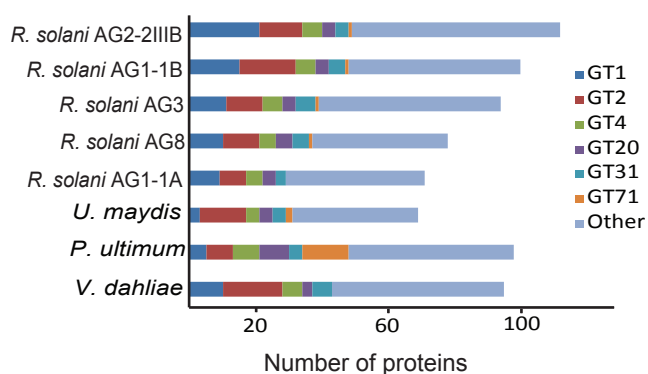

Supplement: Additional file 10: Figure S4. — Number of CAZymes identified in Rhizoctonia solani (five anastomosis groups), Ustilago maydis, Pythium ulticum and Verticillium dahliae. (a) Auxiliary Activities (AA), (b) Carbohydrate Binding Modules (CBM) and (c) Glycosyl Transferases (GT). (PDF 405 kb) [file 12864_2016_2561_MOESM10_ESM.pdf]

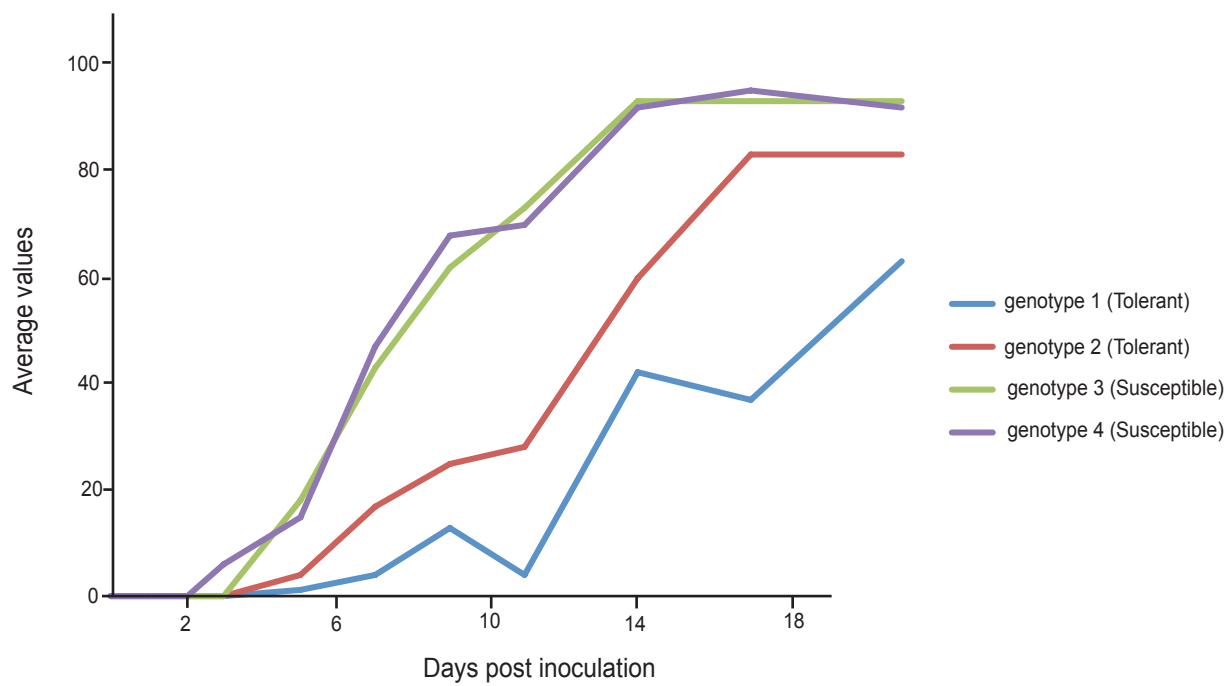

Supplement: Additional file 11: Figure S5. — Phenotypic assessment of R. solani AG2-2IIIB infection on roots of four sugar beet genotypes. 13-weeks old plants, were inoculated with R. solani AG2-2IIIB by putting 4 infected barley kernels approximately 1 cm from the root and 1.5 cm down in the soil on 4 sides of each root. The graph shows estimated percentage of the root surface covered by necrosis in relation to healthy tissue. At least three roots for each time-point were scored. The observations are presented as average values (Y-axis). (PDF 314 kb) [file 12864_2016_2561_MOESM11_ESM.pdf]
