# Supplementary material for: Genome analysis of the sugar beet pathogen Rhizoctonia solani AG2-2IIIB revealed high numbers in secreted proteins and cell wall degrading enzymes
Source: BMC Genomics. 2016 Mar 17;17:245. doi: 10.1186/s12864-016-2561-1 (PMC4794925; doi:10.1186/s12864-016-2561-1)
Supplement: Additional file 8: Figure S2. — Distribution of CAZyme gene families of R. solani AG2-2IIIB within 6 main categories according to the CAZy database. Glysoside hydrolases (GH), Carbohydrate esterases (CE), Auxilliary activity (AA), Carbohydrate-binding modules (CBM), Glycosyl transferases (GT), and Polysaccharide lyases (PL). (PDF 1185 kb) [file 12864_2016_2561_MOESM8_ESM.pdf]

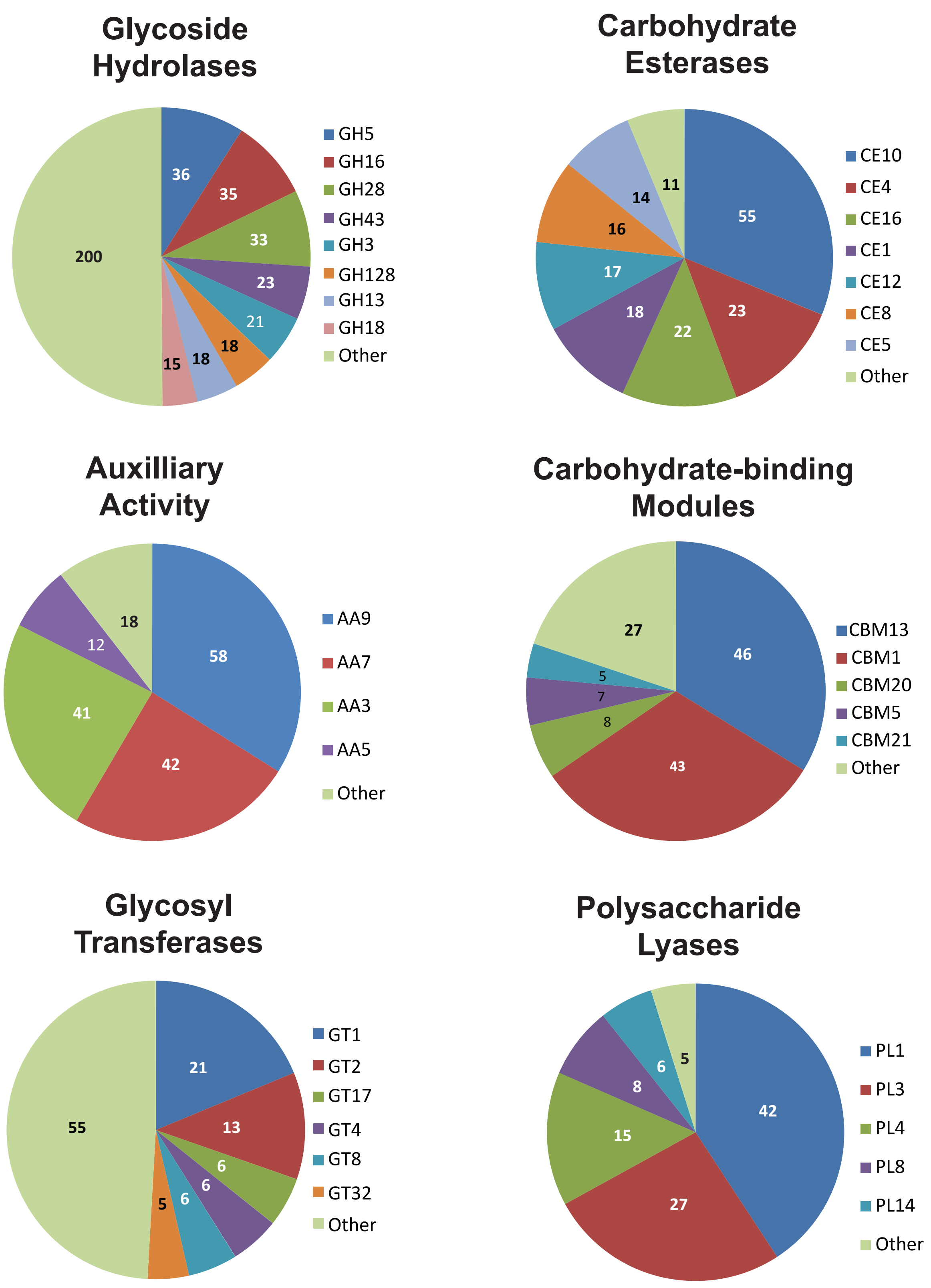

Glycoside Hydrolases
GH5
GH16
GH28
GH43
GH3
GH128
GH13
GH18
Other
36
35
33
23
21
18
18
15
200
Carbohydrate Esterases
CE10
CE4
CE16
CE1
CE12
CE8
CE5
Other
55
23
22
18
17
16
14
11
Auxilliary Activity
AA9
AA7
AA3
AA5
Other
58
42
41
12
18
Carbohydrate-binding Modules
CBM13
CBM1
CBM20
CBM5
CBM21
Other
46
43
8
7
5
27
Glycosyl Transferases
GT1
GT2
GT17
GT4
GT8
GT32
Other
21
13
6
6
6
5
55
Polysaccharide Lyases
PL1
PL3
PL4
PL8
PL14
Other
42
27
15
8
6
5
