# Supplementary material for: Genome analysis of the sugar beet pathogen Rhizoctonia solani AG2-2IIIB revealed high numbers in secreted proteins and cell wall degrading enzymes
Source: BMC Genomics. 2016 Mar 17;17:245. doi: 10.1186/s12864-016-2561-1 (PMC4794925; doi:10.1186/s12864-016-2561-1)
Supplement: Additional file 12: Table S7. — Primer sequences used in the current study. (DOCX 86 kb) [file 12864_2016_2561_MOESM12_ESM.docx]

| **Table S7.** Primer sequences used in the current study | | | | |
| --- | --- | --- | --- | --- |
|  | | | | |
| Gene | **Forward primer 5’-3’** | **Reverse primer 5’-3’** | **Amplicon** | **Annealing Tm** |
| *RSOLAG22IIIB_2432* | ttg cga tgg gaa tgt gtt act ctg | gaa tgc cga gtc cga ggg taa | 101bp | 60°C |
| *RSOLAG22IIIB_11126* | tct ttc act gtt ccc gac gac tgg | cca ccg gta agg cac gat ttg att | 100bp | 60°C |
| *RSOLAG22IIIB_4067* | gtc ctc ccg cag cta cgc ctt ac | cgg gtt gct tgt tct cag gac ttg | 116bp | 60°C |
| *RSOLAG22IIIB_2049* | act cta tca cgc tcg ctc gca act | gcg tgg cca gca ata tca aca aa | 129bp | 60°C |
| *RSOLAG22IIIB_7799* | cgg cgc acg cta caca aa ctg | gat cgg tcg acg ggt cca cat tt | 141bp | 60°C |
| *RSOLAG22IIIB_11200* | tcg cgg gaa acg gag acc aa | cgt ggc ccg tta tgt cgt tga aat | 143bp | 60°C |
| *RSOLAG22IIIB_317* | aca agg ggt cac cat ctc caa taa | cgc gca aac gtg atg gtgt | 122bp | 60°C |
| *RSOLAG22IIIB_8439* | gtt gcg ctc tat ggg ctg att ctt | cgt tgc ctt aat tat gcc gtg gtt | 164bp | 58°C |
| *RSOLAG22IIIB_11504* | gtg ggt gcg gag ctt ctg gtg | cca gga ggt cgt cag ggc agt att | 137bp | 60°C |
| *RSOLAG22IIIB_2417* | ggg ccg ggg gta caa tca ca | cga gtt ccc gac gcc aat aat aga | 160bp | 60°C |
| *RSOLAG22IIIB_1474* | tgc cgc gat tcg tct ggt aaa | ctt cca tcg ctc gtc cca gtgt | 157bp | 60°C |
| *RSOLAG22IIIB_7342* | gta caa caa cgg ccc cat ctt caa | gta acg gcg gac gag gga gag | 125bp | 60°C |
| *RSOLAG22IIIB_7378* | tgc ggc acg agt tgc cat tc | ccc gac ggt agt agg cga ggag | 165bp | 60°C |
| *RSOLAG22IIIB_90* | ccc gga tgg aac gca cgaa | ccg ccc acc cgt cca tctt | 145bp | 58°C |
| *RSOLAG22IIIB_2459* | acc tat ggg gac ccg ttc gttc | tca act tca acc ggg agc cag at | 116bp | 60°C |
| *dpoB* | cag tgg gaa cat gga tag gt | ttg cgt aag aag ttg ttgg | 450bp | 60°C |
| *G3PDH* | acc gtt atgggc ttg tct ttc ctt | ccc gct tgg ctg gaa tag taa cg | 146bp | 60°C |
